# Supplementary material for: Biomass and Leaf Acclimations to Ultraviolet Solar Radiation in Juvenile Plants of Coffea arabica and C. canephora
Source: Plants (Basel). 2021 Mar 28;10(4):640. doi: 10.3390/plants10040640 (PMC8065693; doi:10.3390/plants10040640)
Supplement: Supplementary file 1 [file plants-10-00640-s001.pdf]

Supplementary material

Table S1. Biomass allocation (%) in the leaves, stems, and roots for *Coffea arabica* and *C. canephora* grown under near ambient (UVam) and reduced (UVre) UV levels. Estimated mean values ± S.E. (n = 8) and ANOVA *P*-values of effects of species and UV levels are shown.

Species	UV level	Leaves	Stems	Roots
C. arabica	UVam	42.5 ±1.78	26.2 ±0.90	31.3 ±2.10
	UVre	37.7 ±1.90	29.6 ±0.96	32.7 ±2.25
C. canephora	UVam	37.4 ±1.78	23.3 ±0.90	39.2 ±2.10
	UVre	36.1 ±1.78	23.1 ±0.90	40.8 ±2.10
UV level		0.0671	**0.0192**	0.6276
Species		**0.0443**	**0.0351**	**0.0110**
UV level: species		0.3286	0.0650	0.9787

ANOVA *P*-values < 0.05 are marked in bold, whilst marginal values < 0.1 are underlined.

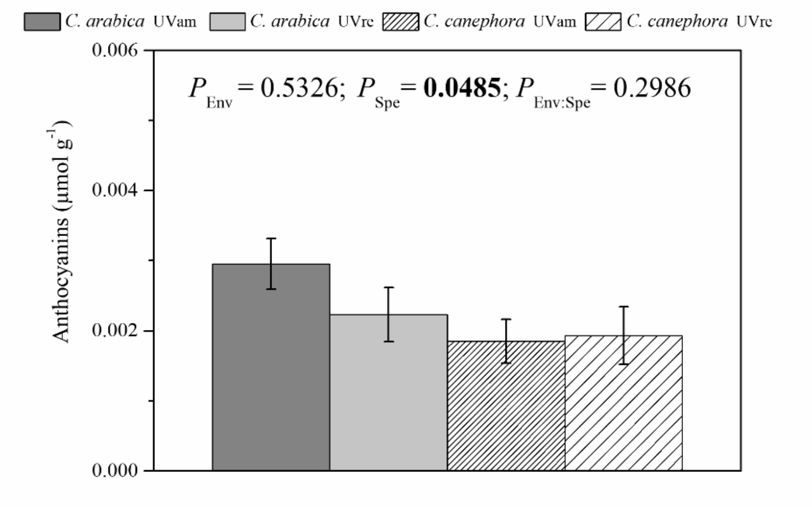

Figure S1: Anthocyanin content for *Coffea arabica* and *C. canephora* (Spe) grown under near ambient (UVam) and reduced (UVre) UV levels (Env). Estimated mean values ± S.E. (n = 8) and ANOVA *P*-values of effects of species and UV levels are shown. ANOVA *P*-values < 0.05 are marked in bold.
